# Examining Exposure to Messaging, Content, and Hate Speech from Partisan News Social Media Posts on Racial and Ethnic Health Disparities

**DOI:** 10.3390/ijerph20043230

**Published:** 2023-02-12

**Authors:** Thu T. Nguyen, Weijun Yu, Junaid S. Merchant, Shaniece Criss, Chris J. Kennedy, Heran Mane, Krishik N. Gowda, Melanie Kim, Ritu Belani, Caitlin F. Blanco, Manvitha Kalachagari, Xiaohe Yue, Vanessa V. Volpe, Amani M. Allen, Yulin Hswen, Quynh C. Nguyen

**Affiliations:** 1 Department of Epidemiology & Biostatistics, University of Maryland School of Public Health, College Park, MD 20742, USA; 2 Department of Health Sciences, Furman University, Greenville, SC 29613, USA; 3 Department of Psychiatry, Harvard Medical School, Boston, MA 02114, USA; 4 Department of Anthropology, Brown University, Providence, RI 02912, USA; 5 Department of Psychology, North Carolina State University, Raleigh, NC 27695, USA; 6 Divisions of Community Health Sciences and Epidemiology, University of California, Berkeley, CA 94704, USA; 7 Department of Epidemiology and Biostatistics, Bakar Computational Health Sciences Institute, University of California San Francisco, San Francisco, CA 94143, USA

**Keywords:** racial health disparities, news media, social media, machine learning

## Abstract

We investigated the content of liberal and conservative news media Facebook posts on race and ethnic health disparities. A total of 3,327,360 liberal and conservative news Facebook posts from the United States (US) from January 2015 to May 2022 were collected from the Crowd Tangle platform and filtered for race and health-related keywords. Qualitative content analysis was conducted on a random sample of 1750 liberal and 1750 conservative posts. Posts were analyzed for a continuum of hate speech using a newly developed method combining faceted Rasch item response theory with deep learning. Across posts referencing Asian, Black, Latinx, Middle Eastern, and immigrants/refugees, liberal news posts had lower hate scores compared to conservative posts. Liberal news posts were more likely to acknowledge and detail the existence of racial/ethnic health disparities, while conservative news posts were more likely to highlight the negative consequences of protests, immigration, and the disenfranchisement of Whites. Facebook posts from liberal and conservative news focus on different themes with fewer discussions of racial inequities in conservative news. Investigating the discourse on race and health in social media news posts may inform our understanding of the public’s exposure to and knowledge of racial health disparities, and policy-level support for ameliorating these disparities.

## 1. Introduction

News media are critical in shaping public knowledge, attitudes, and behaviors and are where a majority of Americans obtain public health information [1,2,3]. Social media is becoming the dominant forum for news media, with nearly half of Americans using social media for sharing and receiving news [4], yet only 36% of social media users fact-check health information seen online with health care professionals [5]. The shifts in how Americans consume and disseminate news have made social media a powerful tool for agenda setting by political parties. This has contributed to the growing divide in messaging about racial health disparities that people are exposed to based on the political ideology of the media they consume [6]. Indeed, it is estimated that 20% of Democrats and 18% of Republicans obtain their news from left- and right-leaning news media bubbles, respectively [7]. Despite the importance of social media for public health messaging, there is little empirical work investigating how racial health disparities are covered by news sources with different political leanings within the social media landscape of the US.

Media coverage has a powerful effect on how people understand public health issues. Differences in public health opinions can be attributed, in part, to how a public health issue is framed, which creates narratives about the issues, their solutions, and perceptions about who is responsible for addressing them [8].

In addition to shaping public discourse, media also contributes to the stigmatization of different groups, which has implications for public health policy [9]. This includes the stigmatization of health conditions and disorders, such as obesity, HIV/AIDS, autism, and substance use disorder [9,10]. Schneider and Ingram’s [11] framework on the social construction of populations proposes that the public is unlikely to support policies to benefit highly stigmatized groups. Indeed, a study found that news media reinforced public stigma toward people with substance use disorders by using dehumanizing labels when covering the opioid epidemic, which contributed to lower support for public health solutions and higher support for punitive approaches [11]. This is only exacerbated by the fact that both left-leaning and right-leaning news organizations use negative emotional content to drive engagement to a similar degree [12], and this is the case even when the news is about something positive [13].

As consumption of health news is becoming increasingly polarized, political affiliation serves to further reinforce these frames. Political affiliation is the greatest predictor of an individual’s awareness of health disparities [14], such that liberals are nearly three times more likely to be aware of racial health inequities than their conservative peers [15]. Beyond influencing popular opinion for different public health solutions, media coverage also influences individuals’ personal embrace of health policies. A study examining the Affordable Care Act found that news media campaigns contributed to increased rates of health insurance coverage [2]. Together, these findings highlight the symbiotic relationship between media coverage and political affiliation in shaping people’s understanding of and attitudes about public health issues.

Despite the influence that media has on public discourse, there are few investigations on the role that social media plays in the coverage of racial and ethnic health disparities in the US. Our study intends to fill this gap by investigating modern communication about public health topics and hate speech directed toward various racial and ethnic groups. Facebook is a dominant source of news for Americans, outpacing other social media platforms [16]. Recent estimates suggest that Facebook is the most used social media platform for obtaining news, with nearly a third of US adults reporting that they obtain their news from Facebook, compared to only 13% of US adults who report obtaining news from Twitter [4]. We analyzed public Facebook posts from conservative and liberal news media organizations using race and health-related keywords. In addition, posts were analyzed for hate speech directed toward various racial and ethnic groups and a qualitative content analysis was conducted to examine thematic content.

## 2. Materials and Methods

Using CrowdTangle, an insights tool made available by Meta [17], we collected public Facebook posts from news media sources across the United States (US). The unique Lists feature of CrowdTangle made it possible to collect posts from news media organizations. We compiled a list of national liberal and conservative news sources that have a significant US-based audience. We classified media outlets according to their audience [18,19] and by a Pew survey that asked people who identify as conservative and liberal the news sources they turn to for news [20]. Foreign news sources such as Great Britain’s The Guardian US and Qatar’s Al Jazeera English were also collected because a substantial number of Americans receive their news from these sources [21]. Using custom-made CrowdTangle lists (Table 1), we collected Facebook posts between January 2015 and May 2022 from US news media pages.

We chose this time period because Facebook’s algorithm was revised in 2015 to flag and reduce the distribution of fake news stories and misleading content [22]. A total of 1,921,820 liberal and 1,405,540 conservative posts were collected and filtered for mentions of race- and health-related keywords. Our race filtering keyword list included over 800 race and ethnicity terms that were classified into eight main categories: Asian, Black, Hispanic, Immigrant, Refugee, Middle Eastern, Native American, and White according to the racial/ethnic or minoritized group the keywords generally reference. While immigrant and refugee are not racial groups, they are minoritized social groups in the US. These keywords were compiled from prior studies examining race-related online conversations [23] and an online database of racial slurs [24] (online supplemental Table S1). Our health-related keyword terms included sick, sickness, health, healthy, well-being, illness, disease, wellness, death, mortality, ill, morbidity, and survival. Our health-related keywords were compiled from the authors. The objective was to capture broad terms related to health and well-being. From the liberal news posts, 145,849 (7.59%) used one or more race-related terms, and 5980 (0.31%) posts used both a race- and health-related keyword. From conservative news, 72,505 (5.16%) posts used one or more race-related terms, and 1750 (0.13%) used both a race- and health-related keyword from 2015–2022. The final analytic sample after filtering for race and health-related terms included 5980 liberal news and 1750 conservative news Facebook posts. 

### 2.1. Hate Speech Measure

Our recently developed method combines faceted item response theory (IRT) with deep learning to measure hate speech on a continuous, interval spectrum. Details on the development of the hate speech measure are documented [25] in previous research. The training dataset, consisting of 50,000 social media comments sourced from YouTube, Twitter, and Reddit, was labeled by 10,000 US-based Amazon Mechanical Turk [26] workers on those components of hate speech (the dataset is available at https://huggingface.co/datasets/ucberkeley-dlab/measuring-hate-speech) (accessed on 21 December 2022) [27]. Amazon Mechanical Turk [26] is a crowdsourcing marketplace where tasks can be performed. The crowdsourced labels were combined via a nonlinear IRT scaling transformation into a continuous outcome measure, yielding an interval-valued spectrum ranging from violent hate speech on one extreme (−5.0) to supportive identity speech on the other (+8.0). The resulting model achieved a cross-validated correlation of 84% and mean absolute error of 0.85 at predicting the continuous hate speech score. This model is more accurate than the 66% correlation and 1.7 mean absolute error of Google Jigsaw’s Perspective API model [28], which is possibly the most widely used hate speech detector. This novel hate speech measurement system allowed us to estimate the precise location of each Facebook post on the hate speech spectrum, where more negative scores were more indicative of violent, racist language, and more positive scores were indicative of benevolent race-related speech. We have recently leveraged this technology to evaluate social media discussions around the 2021 Atlanta Spa shootings [29].

### 2.2. Qualitative Content Analysis

Qualitative content analysis was conducted on a random sample of 1750 liberal posts (out of 5980) and 1750 conservative posts (full sample of conservative news posts collected). The equal number of posts analyzed from conservative and liberal news posts allowed us to compare themes from each type of news source holding the sample size constant. The study team developed the codebook (Table 2) based on reviewing 200 posts from the sample to create codes and definitions. The final codes were (1) US shootings and race relations; (2) immigration; (3) health care and social programs; (4) US health; (5) international health; (6) international news; and (7) US politics.

Using this coding scheme, study members coded the same posts until an inter-rater reliability (Cohen’s Kappa) of 80% or greater was achieved. After each round of coding, the five study team members discussed all discrepancies in the coding and reached consensus on the final codes. After two rounds of coding 200 posts for each round, inter-rater reliability among the coding pairs ranged from 82–93%. Study team members then independently coded the remaining posts. We sought to maintain data trustworthiness through utilizing multiple data analysts with different racial backgrounds and life experiences, and we utilized team meetings to reach consensus.

## 3. Results

**Figure 1:**
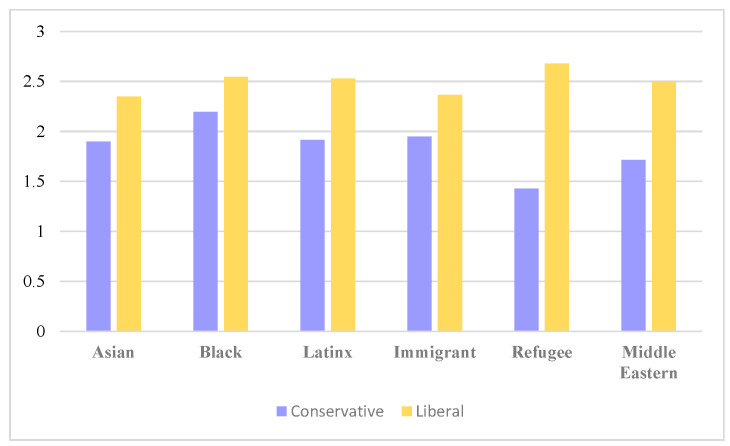
Mean hate score of Facebook posts referencing racial and ethnic groups by conservative and liberal news media. Higher values indicate more supportive benevolent speech, while lower values mean greater hate speech.

Across liberal and conservative news Facebook posts referencing specific racial or ethnic groups, liberal news posts tended to have more positive hate scores, indicating more supportive or benevolent race-related speech, compared to conservative news posts which had more negative hate scores (Figure 1). Liberal news posts were more likely to acknowledge and detail the existence of racial and ethnic health disparities, while conservative news posts were more likely to highlight the negative consequences of Black Lives Matter (BLM) protests, immigration, and the disenfranchisement of Whites.

**Figure 2:**
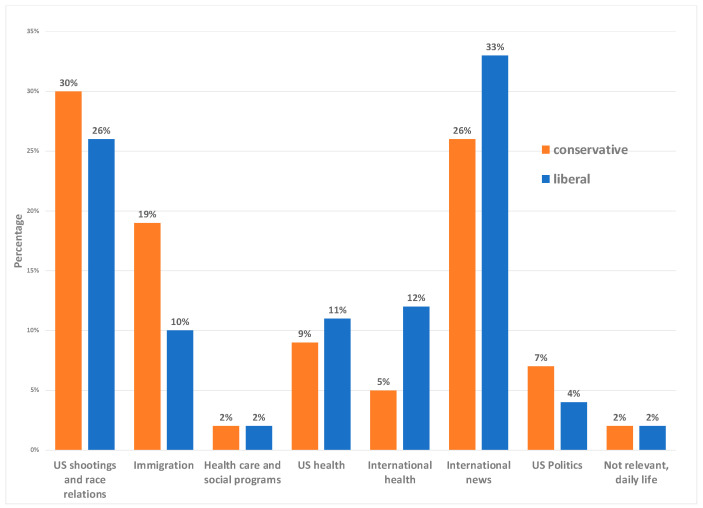
Percent of posts in each content analysis category by conservative and liberal news sources.

Figure 2 presents the percentage of posts in each content analysis category by conservative and liberal news sources. Illustrative news posts are presented in Table 3 Discussions of shootings, social justice, and race relations represented 26% of liberal and 30% conservative news posts (Figure 2). Both liberal and conservative news reported on the shootings and court cases, but liberal news posts were more likely to explicitly identify the race of the shooter (e.g., White police officer). Some posts from liberal news posts discussed systemic racism or the history of racism in the US. Conservative posts touched upon these topics, but they were less frequent. Some conservative news posts offered alternative explanations against systemic bias and suggested a tension between racial justice and patriotism (Table 3). Table 3 presents illustrative examples of the posts by the different themes.

Immigration messages made up 10% of liberal news posts and 19% of conservative news posts (Figure 2). Several liberal news posts had a humanitarian lens related to immigration. For example, these included posts related to the well-being of immigrants and the separation of children at the border. Conservative news posts also used a humanitarian and faith-based lens to discuss the plight of immigrants. For example, one conservative post states:

“Mexican Teen Dies after US Border Agents Told Him to Drink Lethal Liquid Meth…One dead teen and two rogue officers who have been allowed to keep their jobs with minimal consequences. So how is this relevant to Christian news? It’s the injustice that provoked this op-ed. I am even more alarmed that more believers have not taken to social media in outrage that neither Valerie Baird or Adrian Parellon have been disciplined or fired.”

Several conservative news posts also highlighted the negative consequences of immigration to the US. Some conservative news posts highlighted crimes committed by immigrants and the drug crisis, which were used to make a case for stricter immigration policies. Criticisms of current immigration policies were present in both conservative and liberal news posts (Table 3).

Posts related to US race-related health and health disparities made up 9% of conservative news posts and 11% of liberal news posts (Figure 2). Liberal news posts detailed specific health disparities by racial and ethnic group and disease or condition. Some of these posts emphasized structural conditions of inequities that are reproduced and maintained by laws, policies, and practices across various institutions. Research studies investigating these resulting disparities as well as personal stories were highlighted. Conservative news posts also discussed health disparities for people of color, but these were less common. Other conservative health-related posts reframed disparities to emphasize ideas of disenfranchisement and inequities faced by White people and minimized the impact of racism on health and health care. Additionally, some conservative news posts presented arguments against universal health care (Table 3). 

International news made up 5% of liberal and 11% of conservative news posts, respectively (Figure 2). Topics discussed included the COVID-19 pandemic in other countries, illness or death of prominent figures, and terrorism attacks.

## 4. Discussion

Both liberal and conservative news posts mentioning race and health keywords represented a small proportion of all news Facebook posts (0.31% of liberal, 0.13% of conservative), indicating race- and health-related social media news posts are relatively rare. We further analyzed the content of posts using race and health keywords. Analysis using a hate speech measure developed by combining faceted Rasch item response theory with deep learning revealed more supportive speech in liberal news posts mentioning race and health across different racial and ethnic groups referenced compared to conservative Facebook news posts. Through qualitative content analysis, we identified both similarities and differences in the thematic content of these posts. Shootings, social justice, race relations, immigration, and US health and health disparities were emergent themes. Liberal news was more likely to acknowledge structural racism or the history of racism in the US. Discussion of specific health disparities was less common in conservative news posts.

Our findings add to prior research indicating that there is infrequent coverage of racial health disparities in news media [30]. We found discussions of topics related to racial and health inequities were rare in social media posts by news media organizations. This is consistent with work showing a decline in racial health disparity-focused articles published between 1996 and 2005, despite increased emphasis from public health professionals [31]. News media play a crucial role in disseminating public health information and research findings to the public [8,32,33]. What the public considers pressing health issues is shaped by their exposure to media exposure and content [34,35]. The rarity of race and health-related news posts may impact the public’s knowledge about racial health inequities.

Research has identified implications for how racial groups are represented in the coverage of public health matters. For instance, media coverage of the obesity problem in the US has increasingly depicted non-Whites as overweight and obese, which may have impacted public support for obesity prevention efforts [36]. Moreover, a study on media coverage of Trump’s 2016 presidential campaign found an increase in biased speech toward immigration and Latinx individuals, even though there were no real-world drivers of this coverage, such as immigration flow [37]. This study also found that prejudice expressed in media was linked with state-level hate crimes [37]. Thus, the media framing plays an important role in shaping public opinions of marginalized groups, which can have negative consequences for these groups with regard to public policy, violence, and/or discrimination.

Our results provide a snapshot of how the political ideological leaning of news posts relates to the general framing of matters about race and health on social media, with liberal news posts using social, structural, and justice frames, while conservative news posts generally focus on individual responsibility and threats to the safety of White Americans. The consistent use of these frames across a range of issues likely contributes to the liberal–conservative differences in knowledge and perceptions around racial health disparities as well as the differences in how the two groups generally process the news [9,13]. Awareness of the role of racism, discrimination, and other social determinants of health in creating and maintaining health inequities is critical to build consensus and obtain broad support for change [8,34]. We found divergent frames and arguments used in social media posts related to race and health among partisan news. This may be a barrier to achieving consensus with both understanding the problem and the solutions needed to address those problems. There is limited prior research exploring and quantifying racial and health discussions from news media with a partisan preference. Our study implemented an analysis of supportive and hate speech using a machine learning model and qualitative content analysis to advance our understanding of how racial health disparities could be expressed and framed in different ways between conservative and liberal news social media posts and, therefore, inform public attitudes and perceptions of racial health disparities. 

There are important limitations to note. We collected posts based on specific keywords. This list is not exhaustive, but we attempted to be as comprehensive as possible and included a range of keywords from neutral terms to slurs. Additionally, it should be noted that keywords referencing racial/ethnic or social groups change over time. Changing the keyword list may yield different results. We collected social media posts from a list of conservative and liberal news organizations. This list was not exhaustive, and there are news organizations with conservative and liberal audiences that are not included on this list. The social media list also does not include non-partisan news. News media organizations also communicate to the public via television, radio, and full-length articles. However, communication and information sharing through social media represent a growing source of information for the public.

## 5. Conclusions

This study provides insights about the sparsity of coverage on social media on topics related to racial health disparities by partisan news. Investigating the discourse on race and health in news posts may inform our understanding of the public’s exposure to and awareness of racial and ethnic health inequities. The tone and framing of the social media message by news media may impact support for policies, programs, and practices aimed at reducing these disparities. Importantly, we characterized the content of conversations by liberal and conservative news sources and identified key distinguishing factors. Our findings are important as approximately one fifth of Republicans and Democrats are in a news bubble and only obtain news from news media outlets predominantly aligned with their party [7]. The reduced coverage of racism- and discrimination-related content in conservative news provides an opportunity for community members, scholars, and activists to engage with conservative news sources to promote content that showcases the various forms of racism that currently impact people across the United States. Additionally, while liberal news sources provide content on health disparities and experiences of racism and discrimination, the coverage is also low and could be bolstered to reflect the experiences of large segments of the population whose well-being and access to opportunities are impacted.

## Figures and Tables

**Table 1:** List of conservative and liberal news media included in the study sample.

Conservative News Media	Liberal News Media
American Thinker	ABC News
CBN News	Al Jazeera English
CNSNews.com	BuzzFeed
Conservative Review	CBS News
Daily Wire	CNN
Fox News	Daily Show
Hot Air	Guardian US
National Review	Huffington Post
New York Post	MSNBC
Newsmax	NBC
PJ Media	New York Times
Red State	NPR
The American Conservative	PBS
The American Spectator	Politico
The Blaze	Slate
The Daily Caller	The Economist
The Daily Signal	The NewYorker
The Federalist	Washington Post
The Heritage Foundation	
The Washington Times	
The Western Journal	
Townhall.com	
Twitchy	
Washington Examiner	
Washington Free Beacon	

**Table 2:** Qualitative codebook.

Themes	Subthemes
1. US shootings, social justice, race relations	1. Shootings (including court case decisions)
	2. Race relations or racism-related (includes Muslims, Jewish people, Christianity, etc.)
2. Immigration	1. Mentions US politics (includes politicians, elections, political issues such as “The Wall”).
	2. Mentions health/health care/social program (directly mentions health care for immigrants)
3. Health care insurance and/or social programs	
4. Health (outbreaks, life expectancy, death, infectious diseases, chronic conditions)	1. US health disparities (includes public health prevention, treatment, and intervention).
	2. International health
5. International news	1. Terrorism
	2. Death (includes public figures)
6. US politics/government/elections (does not mention immigration/migration)	
99. Not relevant/daily life	

**Table 3:** Content analysis of themes with illustrative examples by liberal and conservative news.

Categories	Liberal Themes	Conservative Themes
**Discussions of shootings of African Americans, social justice, and race relations**	*Discussion of shootings and cases* In July 2014 an unarmed black man named Eric Garner died at the hands of a police officer after allegedly resisting arrest. His death, along with that of other unarmed black men accused of petty offenses by white police officers, has raised questions about police tactics. The months-long set of trials for the six Baltimore police officers charged in the death of Freddie Gray are being watched as a rare opportunity for accountability in police brutality, disproportionately against unarmed African Americans. *Identification of the shooter (their race/ethnicity, specifically being White)* Police in St. Louis arrested over a dozen people amid protests after a White former police officer was found not guilty of murder in the 2011 death of a black man who was shot five times in his car after a high-speed chase. The family of an unarmed black man fatally shot by a White Oklahoma officer filed a wrongful death lawsuit in federal court against the city of Tulsa and the policewoman, who was acquitted of manslaughter charges last month. *Structural racism/a long history of racism* This death is not new. Black death at the hands of the state, at the hands of police, at the hands of racist vigilantes is not new. It is a part of the blood and the structure of this country. In the past twenty-five years, it has become increasingly apparent that, while much of the country has evolved beyond the death penalty, the states that remain most committed to it are those that once practiced slavery. Dr. Marcella Alsan, a physician and economist, studies how systemic racism contributes to a lack of trust that perpetuates negative health outcomes in communities of color. Learn more with PBS NewsHour.	*Discussion of shootings and cases* The Tamir Rice verdict sparked outrage from celebrities, activists and politicians after it was announced Monday that Cleveland police would not go to trial in the death of a 12-year-old boy they fatally shot. *Backlash or Countermovement to Black Lives Matter* This officer would have rather been beaten to death than protect herself. Way to go, # BlackLivesMatter. Black Lives Matter protesters are filling the streets of Chicago Wednesday, blocking traffic and disrupting the city to protest police actions relating to the death of 17-year-old Laquan McDonald. Black Lives Matter protest blocks ambulance with sick child headed for hospital.White House National Security Adviser Robert O’Brien said Sunday he doesn’t think there is systemic racism across the nation’s police forces as violent protests erupt in the wake of George Floyd’s death. *Appropriate terms to undermine* MSNBC continues to play the race card and call “racism” that other countries are trying to help Ukrainians fleeing daily shelling and death. Former Baltimore Police Commissioner Anthony Batts said that officers “took a knee” following the death of Freddie Gray, allowing crime to spike because they felt a lack of support from police and city officials. *Questioning or denial of racism* After Freddie Gray’s death in 2015 national conversations began over the legacy of racism in Baltimore. Most of these conversations failed to note that at the time of the incident, the mayor of Baltimore was black; the majority of the city council was black; the police chief was black; the prosecutor against the police was black; three of the charged officers in Gray’s case were black; the congressman for the district was black; the president of the United States was black; and the attorney general of the United States was black. In the wake of the George Floyd death, more and more school districts are adding critical race theory, which teaches that America and whites are inherently racist to their course requirements for graduation. American parents are SICK of critical race theory being taught to their kids as truth in school, according to a new poll…[name] to break down what he found and how far parents including Democrats and Independents are willing to go to protect their children from. There goes CNN again, linking the GOP to racism. How sick are you of this garbage? *Racism, nationalism, patriotism* The media’s thrilled about bagging Schnatter because they’ve strengthened the narrative that patriotic Americans are just white nationalists waiting to be unmasked. Never forget: Schnatter wasn’t taken down over racism (there wasn’t any). He was taken down to make patriotic Americans look racist. And that’s incredibly sick. Black Lives Matter protesters burned an American flag Wednesday night after the announcement that no officers would be charged in the death of Jamar Clark.
**Immigration**	*Justice and humanitarian lens to discuss immigration* US Dept. of Health & Human Services (HHS) officials refuse to answer when asked if the agency is still receiving migrant children who were separated from their parents, but 2047 separated children are still in their custody. Citing a “troubling pattern of abuse and poor treatment” of migrants—after the death of a 16-year-old Guatemalan detainee and other revelations—the House Oversight and Reform Committee demanded documents from the Department of Homeland Security as part of an investigation. By citing a public health law, US officials at the southern border have been able to expel migrants to Mexico or their home countries without allowing them to apply for humanitarian refuge. *Health and immigrants* Sexual and reproductive health is a rarely discussed aspect of migrant women’s vulnerabilities. *Criticism of Trump’s immigration policies* Donald Trump has been talking about building a wall between the US and Mexico since he launched his presidential campaign—but there’s been some confusion over what he’s promised. According to the latest NBC News/Wall Street Journal poll, Americans say Democrats are better at dealing with immigration. News Analysis: President Trump’s 17 months in office have in fact been an exercise in futility for the art-of-the-deal president. No deal on immigration… In one day, the Trump admin. announced it would weaken the Endangered Species Act and set new standards for immigrants to meet, such as having a good credit score and private health insurance. Rachel Maddow: “On the same day, targeting bald eagles and the Statue of Liberty, because America.”	*Immigration as a threat to the US* The drug crisis is an immigration problem, not a health care problem. Wisdom demands we recognize immigration as the most dangerous threat to America’s survival since the Cold War. Border security is a life-or-death issue for Americans *Highlight crimes committed by immigrants as argument for stricter immigration policies* The disturbing verdict in the killing of Kate Steinle finding a criminal illegal alien not guilty of the most serious charges for her death illustrates the need to find a way to end the sanctuary cities movement that has spread around the nation. *Criticism of immigration-related policies* The Biden administration will remove Title 42, a public health rule that has stopped millions of illegal immigrants from crossing the border, on May 23. And it’s already warning that a flood of border crossings will come soon after. But, [name] warns, that may only be the start of our problems: “We’re giving all of the enemies of the United States plenty of notice that it’s going to be chaos at our border that weekend. When Obamacare was written it was designed so that illegal immigrants wouldn’t get federally subsidized health care, but counties across the country still have programs providing care to uninsured illegals.
**Health and health care**	*Health disparities, racism, and social factors impacting health and health care* *Research Studies* Female patients and people of color are more likely to have their symptoms dismissed by medical providers, studies show. [name] readers shared their experiences with what many refer to as “medical gaslighting.” Health care disparities among blacks and Latinos compared to whites has narrowed because of the Affordable Care Act, also known as “Obamacare,” according to a new study. Heart disease is a leading cause of death, but non-Hispanic black people are more than twice as likely to die of it. *Personal stories* Medical student Alejandra Duran Arreola dreams of becoming an OB-GYN in her home state of Georgia, where there’s a shortage of doctors and one of the highest maternal mortality rates in the US But the 26-year-old Mexican immigrant’s goal is now trapped in the debate over the DACA program. *Under-representation* Ethnic and gender diversity among health care providers can increase the depth and scope of information patients are willing to share in clinical settings—information that’s important to their health. And yet, despite rapid growth in the field, the number of African Americans, Hispanics and Native Americans working as genetic counselors has remained low. African Americans and Latinos are especially vulnerable to Alzheimer’s, yet they’re often underrepresented in scientific studies of the disease. A nationwide effort is trying to change that.	*Racial/ethnic health disparities* The coronavirus has disproportionately affected the Latino community as well as the black community according to data from the Centers for Disease Control. The COVID-19 pandemic has hit minority communities particularly hard. Available data indicate that death rates of Hispanic persons are 164% higher than the death rates for white persons. In US communities with high levels of racial prejudice, both blacks and whites may have worse survival odds than people who live in more tolerant places. *Arguments against universal health care* There is a reason New York City’s hospitals are full of rich Canadians who cannot afford the free health care at home. If more money and more direction from Washington isn’t the answer, and neither is putting everyone in a European-style national health care system, what is? What if we tried the completely opposite approach and put individuals and patients, also known as consumers, in the driver’s seat. *Minimization of racism* Contrary to claims that the proclamation constitutes a racist attack on a community who deserves health care, the order says not a word about a specific race, or national or ethnic group. ‘China virus’ ‘Wuhan virus’ While he should focus on health and pandemic relief, the Biden team is too worried about words. Racial discrimination and racism could have died a well-deserved death, but it’s been resurrected by poverty pimps and other people who benefit from exploiting the problems that black people face in our country. *Health disparities for white people* The death rate for white women in rural areas has been on the rise since 2000. White people who have the same risk factors are not given the same priority ranking for the antibody treatments as non-White people who become ill from coronavirus. *Religion and the church* Statistics show half of all men and a third of all women will hear these words -- you have cancer. Churches should be a sanctuary for support. However, in many cases, people suffer in silence as they cope with their illness. Now the Cancer Treatment Centers of America is offering a free program to break down the wall of silence. They’re teaching pastors how to minister effectively to cancer patients. Mexican Teen Dies after US Border Agents Told Him to Drink Lethal Liquid Meth…One dead teen and two rogue officers who have been allowed to keep their jobs with minimal consequences. So how is this relevant to Christian news? It’s the injustice that provoked this op-ed. I am even more alarmed that more believers have not taken to social media in outrage that neither Valerie Baird or Adrian Parellon have been disciplined or fired. Proverbs 1:16–19 outlines the six things God hates…
Some posts were edited or shortened to remove identifying information. Hashtags, urls, and tags were removed. International news examples were not included because they were not directly related to US discussions on race and health.		

## Data Availability

The data that support the findings of this study are available from Facebook posts collected through CrowdTangle. CrowdTangle is a public insights tool from Meta that enables users to follow, analyze, and report on what is happening with public content on social media.

## Supplementary Materials

The following supporting information can be downloaded at: https://www.mdpi.com/article/10.3390/ijerph20043230/s1, Table S1: Race, ethnicity, and minoritized social group terms used in data collection.
