# Supplementary material for: Examining Exposure to Messaging, Content, and Hate Speech from Partisan News Social Media Posts on Racial and Ethnic Health Disparities
Source: Int J Environ Res Public Health. 2023 Feb 12;20(4):3230. doi: 10.3390/ijerph20043230 (PMC9960309; doi:10.3390/ijerph20043230)
Supplement: Supplementary file 1 [file ijerph-20-03230-s001.zip › ijerph-2182367-supplementary.pdf]

Online Supplementary Materials

Table S1. Race, ethnicity, and minoritized social group terms used in data collection.

Items	Category
aapi	asian
abg's	asian
chinese	asian
afghans	asian
almond shaped eyes	asian
an abg	asian
asian	asian
asian indian	asian
asians	asian
aznbbygirl	asian
bamboo coon	asian
bangalees	asian
bangladeshi	asian
bangladeshi	asian
bengalis	asian
buddha	asian
buddhahead	asian
buddhas	asian
burmese	asian
cambodian	asian
cambodians	asian
chiegro	asian
chinaman	asian
asian	asian
asian	asian
asians	asian
ching chong	asian
ching-chong	asian
chink	asian
chinks	asian
chinky	asian
chonky	asian
coconut nigger	asian
coolie	asian
cracker jap	asian
cunt-eyed	asian
dink	asian
dog muncher	asian
dog-muncher	asian
dothead	asian
east asian	asian
filipino	asian
filipinos	asian
fingernail rancher	asian
fresh off	asian
ganesha	asian
gook	asian
gookaniese	asian

gookemon	asian
gooky	asian
gyppo	asian
hawaiianculture	asian
hindi	asian
hindu	asian
hinduism	asian
hindus	asian
hindutva	asian
huns	asian
indonesian	asian
jap	asian
japs	asian
japanese	asian
kashmiri	asian
kashmiris	asian
korean	asian
koreans	asian
koreanstyle	asian
koreas	asian
lao	asian
laotian	asian
ling ling	asian
little hiroshima	asian
malayali	asian
malaysian	asian
maori	asian
mongol	asian
mongolian	asian
mongolians	asian
mongols	asian
nepal	asian
nepalese	asian
noodle nigger	asian
north korean	asian
oriental	asian
orientals	asian
asian	asian
pajeet	asian
paki	asian
pashtuns	asian
philippine	asian
polynesian	asian
polynesians	asian
punjab	asian
pyongyang	asian
rice burner	asian
rice nigger	asian
rice rocket	asian
rice-nigger	asian
rohingya	asian
samoan	asian
sideways cooter	asian
sideways pussy	asian

sikhs	asian
slanted eye	asian
slant-eye	asian
slanty eye	asian
slanty eyed	asian
slopehead	asian
south asian	asian
squinty	asian
sumerians	asian
tagalog	asian
taiwanese	asian
tamil	asian
teaceremony	asian
thai	asian
thais	asian
thin eyed	asian
thineyed	asian
thin-eyed	asian
tibetan	asian
currymuncher	asian
uyghurs	asian
vietcong	asian
vietnamese	asian
zipperhead	asian
wuhanvirus	asian
wuhan virus	asian
china virus	asian
chinavirus	asian
asianvirus	asian
asian virus	asian
kungflu	asian
kung flu	asian
yellow peril	asian
commie cough	asian
wuhanic plague	asian
madcau disease	asian
chingchongvirus	asian
kungflufighting	asian
kungfuflu	asian
chingchongprague	asian
commiecough	asian
wuflu	asian
chinadisease	asian
prayforchina	asian
blamechina	asian
wuhanpneumonia	asian
itschinasfault	asian
chinacorona	asian
hindoo	asian
pacific islander	asian
chamorro	asian
guamanian	asian
#blacklivesmatter	black
#blktwiter	black

#tamirrice	black
african american	black
african americans	black
african't	black
africoon	black
afro caribbean	black
afro-caribbean	black
atatianajefferson	black
atatiana jefferson	black
backtheblue	black
banjo lip	black
bantuknots	black
biscuit lip	black
bix nood	black
black ppl	black
black people	black
black boy	black
black boys	black
black female	black
black girl	black
black girls	black
black history	black
black lives	black
black male	black
black man	black
black men	black
black panther	black
black twitter	black
black woman	black
black women	black
blackeconomics	black
blackexcellence	black
blackgirlmagic	black
blackgirls	black
blackisbeautiful	black
blackity	black
blacklivesmatter	black
blacklove	black
blackpeople	black
blackpride	black
blacks	black
blacktwitter	black
blackunity	black
blackwomen	black
blks	black
blktwiter	black
blktwitter	black
blm	black
blue lives	black
bluelivesmatter	black
bootlip	black
buffie	black
bumper lip	black
burnt cracker	black

burrhead	black
bush-boogie	black
carribean people	black
chain dragger	black
congo lip	black
congolese	black
coon	black
coonass	black
coon-ass	black
coontang	black
dark skin	black
darkskin	black
darkey	black
darkie	black
darky	black
drc	black
ebennettart	black
ericgarner	black
eric garner	black
ethiopian	black
ethiopians	black
field nigger	black
freddie gray	black
freddiegray	black
golliwog	black
groid	black
haitians	black
hotep	black
jamaican	black
jamaicans	black
jigarooni	black
jigga	black
jiggabo	black
jigger	black
jim crow	black
knuckle-dragger	black
koon	black
light skin	black
lightskin	black
mandinka	black
moulie	black
mud people	black
n word	black
negro	black
negroes	black
negros	black
nevisian	Black
nigette	black
nigga	black
niggah	black
niggas	black
nigger	black
niggers	black
nigglet	black

nigglets	black
niglet	black
nig-ngo	black
nsbe	black
n-word	black
pickaninny	black
policebrutality	black
porch monkey	black
sandrabland	black
sandra bland	black
sayhername	black
shopblack	black
slavery	black
slaves	black
somali	black
spearchucker	black
staywoke	black
take a knee	black
takeaknee	black
tamir + rice	black
tar baby	black
tar-baby	black
themelaninleague	black
africans	black
bahamas	black
bahamian	black
bahamians	black
bantu	black
blackcommunity	black
blackpanther	black
burundi	black
cameroon	black
caribbeans	black
congo	black
cuban	black
cubano	black
cubans	black
dominican	black
ghanaian	black
haitian	black
jigaboo	black
kenyan	black
liberian	black
mozambican	black
nigeria	black
nigerian	black
nigerians	black
niggress	black
nubians	black
rwandan people	black
shona	black
somalian	black
somalis	black
south african	black

sudan	black
sudanese	black
swahili	black
tanzania	black
tanzanian	black
usorelse	black
zambian	black
zimbabwean	black
new mexico border	exclude
beaner	hispanic
border bandit	hispanic
border control	hispanic
border fence	hispanic
border hopper	hispanic
border jumper	hispanic
border nigger	hispanic
border security	hispanic
border surveillance	hispanic
border wall	hispanic
boricua	hispanic
brazilians	hispanic
build a wall	hispanic
buildawall	hispanic
buildourwall	hispanic
buildthatwall	hispanic
buildthedamnwall	hispanic
buildthewall	hispanic
buildthewallnow	hispanic
central american	hispanic
chexican	hispanic
chicano	hispanic
chicanos	hispanic
chicanotattoo	hispanic
chicanx	hispanic
colombian	hispanic
colombians	hispanic
columbians	hispanic
costa rican	hispanic
dampback	hispanic
dayofthedead	hispanic
domican	hispanic
dominicans	hispanic
ecuadorian	hispanic
fence fairy	hispanic
fence hopper	hispanic
fence-hopper	hispanic
fesskin	hispanic
finishthewall	hispanic
growinguphispanic	hispanic
guatemalans	hispanic
hispandex	hispanic
hispanic	hispanic
hispanicheritagemonth	hispanic
hispanics	hispanic

hispano	hispanic
hondurans	hispanic
latina	hispanic
latinas	hispanic
latinbeauty	hispanic
latino	hispanic
latinos	hispanic
latins	hispanic
latinx	hispanic
latinxs	hispanic
mayans	hispanic
mex	hispanic
mexcrement	hispanic
mexican	hispanic
mexicancocina	hispanic
mexicanos	hispanic
mexicans	hispanic
mexican't	hispanic
mexico border	hispanic
mexicoborder	hispanic
mexicocity	hispanic
mexicoon	hispanic
mexihos	hispanic
nicaraguans	hispanic
panamanian	hispanic
peruvian	hispanic
puerto rican	hispanic
puerto ricans	hispanic
raza	hispanic
salsacolombiana	hispanic
salvadorans	hispanic
salvadoreans	hispanic
salvadorians	hispanic
secureourborder	hispanic
south american	hispanic
spic	hispanic
spick	hispanic
spig	hispanic
spigotty	hispanic
spik	hispanic
taco nigger	hispanic
tamaulipas	hispanic
thicklatina	hispanic
venezuelans	hispanic
wetback	hispanic
wetblack	hispanic
deport	immigrant
deportation	immigrant
deported	immigrant
deporting	immigrant
deports	immigrant
end sanctuary	immigrant
first generation immigrant	immigrant
foreigner	immigrant

foreigners	immigrant
go back where	immigrant
gobackwhere	immigrant
illegal alien	immigrant
illegal aliens	immigrant
illegal immigrant	immigrant
illegal immigrants	immigrant
illegalalien	immigrant
illegalaliens	immigrant
illegalimmigrants	immigrant
illegals	immigrant
imigrant	immigrant
imigration	immigrant
immagrant	immigrant
immagration	immigrant
immig	immigrant
immigrant	immigrant
immigrants	immigrant
immigrantion	immigrant
immigrants	immigrant
immigrates	immigrant
immigration	immigrant
immigrations	immigrant
immingrant	immigrant
migrant	immigrant
migrants	immigrant
naturalized	immigrant
openborders	immigrant
our country back	immigrant
ourcountryback	immigrant
sanctuary	immigrant
sanctuary cities	immigrant
sanctuary city	immigrant
sanctuarycities	immigrant
sanctuarycity	immigrant
sanctuarystate	immigrant
sanctuarystates	immigrant
second generation immigrant	immigrant
secure our border	immigrant
travel ban	immigrant
undocumented	immigrant
afganistan	middle eastern
afghanistan	middle eastern
afghanistani	middle eastern
afghanistans	middle eastern
andsf	middle eastern
arab	middle eastern
arabs	middle eastern
arabush	middle eastern
armenians	middle eastern
baghdad	middle eastern
ban islam	middle eastern
ban muslim	middle eastern
ban on muslims	middle eastern

banislam	middle eastern
banmuslim	middle eastern
banonmulsims	middle eastern
cairo coon	middle eastern
camel cowboy	middle eastern
camel fucker	middle eastern
camel jacker	middle eastern
camelfucker	middle eastern
camel-fucker	middle eastern
cameljacker	middle eastern
camel-jacker	middle eastern
canaanites	middle eastern
carpet pilot	middle eastern
carpetpilot	middle eastern
clit chopper	middle eastern
clit-chopper	middle eastern
clitless	middle eastern
clit-swiper	middle eastern
derka derka	middle eastern
derkaderka	middle eastern
diaper head	middle eastern
diaperhead	middle eastern
diaper-head	middle eastern
dune coon	middle eastern
dune nigger	middle eastern
dunecoon	middle eastern
dunenigger	middle eastern
durka durka	middle eastern
durka-durka	middle eastern
egyptians	middle eastern
freemiddle eastern	middle eastern
fuckmuslims	middle eastern
hambaya	middle eastern
hebrews	middle eastern
hijab	middle eastern
hijabi	middle eastern
hijabs	middle eastern
ilan	middle eastern
irani	middle eastern
iranian	middle eastern
irans	middle eastern
iraqs	middle eastern
irgc	middle eastern
islamist	middle eastern
islamists	middle eastern
islams	middle eastern
israels	middle eastern
jig-abdul	middle eastern
jihad	middle eastern
jihadi	middle eastern
jihadis	middle eastern
jihadist	middle eastern
jihads	middle eastern
kaaba	middle eastern

kafeir	middle eastern
kuffar	middle eastern
middle eastern	middle eastern
moroccan	middle eastern
moslem	middle eastern
mudshark	middle eastern
muhammadalijinnah	middle eastern
muslim	middle eastern
muslimban	middle eastern
muslin	middle eastern
muzrat	middle eastern
muzzie	middle eastern
operationpeacespring	middle eastern
pashtun	middle eastern
pegida	middle eastern
peshmerga	middle eastern
phoenicians	middle eastern
pisslam	middle eastern
qtip head	middle eastern
rag head	middle eastern
raghead	middle eastern
rapefugee	middle eastern
rug pilot	middle eastern
rug rider	middle eastern
rugpilot	middle eastern
sand flea	middle eastern
sand monkey	middle eastern
sand moolie	middle eastern
sand nigger	middle eastern
sand rat	middle eastern
sandflea	middle eastern
sandmonkey	middle eastern
sandmoolie	middle eastern
sandnigger	middle eastern
sandrat	middle eastern
saudis	middle eastern
shiptar	middle eastern
slurpee nigger	middle eastern
slurpeenigger	middle eastern
sun goblin	middle eastern
sunnis	middle eastern
syrias	middle eastern
tehran	middle eastern
turks	middle eastern
wahabi	middle eastern
whacky iraqi	middle eastern
whitegenocide	middle eastern
yemen	middle eastern
afg	middle eastern
afghan	middle eastern
al qaeda	middle eastern
al-qaeda	middle eastern
arabian	middle eastern
arabic	middle eastern

aramaic	middle eastern
asslifter	middle eastern
assyrian	middle eastern
badghis	middle eastern
baloch	middle eastern
bansharialaw	middle eastern
bhutanese	middle eastern
carpet kisser	middle eastern
death to islam	middle eastern
egyptian	middle eastern
farsi	middle eastern
goat fucker	middle eastern
haji	middle eastern
hajji	middle eastern
hamas	middle eastern
hojabi	middle eastern
impeachilhanomar	middle eastern
iranians	middle eastern
iraqi	middle eastern
iraqis	middle eastern
islam	middle eastern
islamic	middle eastern
islamics	middle eastern
israeli	middle eastern
israelis	middle eastern
isreali	middle eastern
jehad	middle eastern
jordanian	middle eastern
koranimal	middle eastern
kurd	middle eastern
kurdish	middle eastern
kurds	middle eastern
lebanese	middle eastern
lebanese	middle eastern
levantines	middle eastern
malayer	middle eastern
middle eastern	middle eastern
moosrats	middle eastern
moroccans	middle eastern
moslems	middle eastern
mud slum	middle eastern
mudslum	middle eastern
muslims	middle eastern
muzzies	middle eastern
muzzrats	middle eastern
middle eastern	middle eastern
palestinian	middle eastern
palestinians	middle eastern
persian	middle eastern
persian	middle eastern
persians	middle eastern
shias	middle eastern
syrian	middle eastern
syrianrefugee	middle eastern

syrians	middle eastern
taliban	middle eastern
taliban	middle eastern
tunisians	middle eastern
turkish	middle eastern
urdu	middle eastern
yazidi	middle eastern
yemeni	middle eastern
yiddish	middle eastern
bipoc	minority
brown people	minority
brownskin	minority
brown skin	minority
ethnics	minority
minorities	minority
myhousemyamerica	minority
people of color	minority
person of color	minority
poc	minority
pocs	minority
race traitor	minority
racism	minority
racist	minority
racists	minority
woc	minority
wog	minority
beaner shnitzel	multi-race
beanershnitzel	multi-race
biracial	multi-race
caublasian	multi-race
half breed	multi-race
half cast	multi-race
half-breed	multi-race
half-cast	multi-race
interracial	multi-race
intraracially	multi-race
bow bender	native american
buffalo jockey	native american
bushnigger	native american
cherry nigger	native american
hatchet-packer	native american
prairie nigger	native american
red nigger	native american
river nigger	native american
rivernigger	native american
squaw	native american
teepee creeper	native american
tee-pee creeper	native american
timber nigger	native american
timbernigger	native american
tomahawk chucker	native american
tomahawk-chucker	native american
tomahonky	native american
american indian	native american

apache	native american
apache indian	native american
apache nation	native american
apache tribe	native american
cherokee	native american
cherokee indian	native american
cherokee nation	native american
cherokee tribe	native american
chippewa indian	native american
chippewa nation	native american
chippewa tribe	native american
choctaw	native american
choctaw indian	native american
choctaw nation	native american
choctaw tribe	native american
hula	native american
iroquois indian	native american
iroquois nation	native american
iroquois tribe	native american
luau	native american
native american	native american
native americans	native american
native hawaiian	native american
navajo	native american
navajostrong	native american
navajoweavingrules	native american
pueblo indians	native american
pueblo nation	native american
pueblo tribe	native american
sioux	native american
sioux indian	native american
sioux nation	native american
sioux tribe	native american
tohono	native american
aid refugee	refugee
asylee	refugee
help refugee	refugee
migrant protection program	refugee
norefugeeban	refugee
refugee	refugee
refugeelivesmatter	refugee
refugee	refugee
refuges	refugee
resettlement	refugee
unhcr	refugee
we welcome refugee	refugee
welcome refugee	refugee
welcomerefugee	refugee
all lives	white
alllivesmatter	white
anglo	white
aryan	white
aryans	white
gringo	white

gringos	white
hillbilly	white
hilljack	white
hillwilliam	white
honkey	white
honkeys	white
honky	white
honkys	white
karen people	white
kkk	white
klanswoman	white
nazis	white
qwhite	white
romans	white
skinhead	white
skinheads	white
trailer trash	white
whigger	white
white guy	white
white people	white
white ppl	white
white boy	white
white boys	white
white devil	white
white girl	white
white girls	white
white man	white
white men	white
white trash	white
white woman	white
white women	white
whitetrash	white
whitey	white
whiteys	white
alllifematters	white
anglo	white
aryan	white
blue eye devil	white
buckra	white
caucasian	white
caucasians	white
colonialists	white
gringo	white
guido	white
hillbillies	white
hillbillys	white
klansman	white
oath keepers	white
oathkeepers	white
proud boys	white
redneck	white
saxon	white
white flight	white
whites	white

wigga	white
wigger	white
wypipo	white
